# Intraluminal Monitoring of Micro Vessels. A Surgical Feasibility Study

**DOI:** 10.3389/fsurg.2021.681797

**Published:** 2021-07-21

**Authors:** Leonard Walle, Holger Sudhoff, Onno Frerichs, Ingo Todt

**Affiliations:** ^1^Department of Otolaryngology, Head and Neck Surgery, Medical School OWL, Bielefeld University, Bielefeld, Germany; ^2^Department of Plastic Surgery, Medical School OWL, Bielefeld University, Bielefeld, Germany

**Keywords:** microvessel, pressure monitoring, fiber optic pressure measurement, anastomosis, flap monitoring device

## Abstract

**Objective:** Monitoring of vessel perfusion is of high clinical importance in vascular anastomosis of free flaps. Current sensor systems are based on different principles and show limitations in validity and accuracy. Fiber optic pressure sensors exhibit high accuracy and are small in size. The aim of the present study was to evaluate the surgical feasibility of intraluminal pressure (ILP) measurements with a fiber optic pressure sensor in an animal model.

**Methods:** In a microsurgical setting we sedated 10 Wistar rats with weight adapted phenobarbital, xylazine, and fentanyl. We performed a surgical approach to A. carotis communis and V. jugularis and introduced a 600 μm fiber optic pressure sensor into the vessels followed by measuring the ILP. The sensor was stabilized by the surrounding tissue, and the vessels were closed.

**Results:** In all cases, surgical placement was uneventful. Measurement of intra-venous and intra-arterial pressure was possible and stable over the whole measurement period of an hour.

**Conclusion:** Fiber optic pressure measurement in microvessels is possible and surgically feasible. An application to monitor the perfusion of free flaps seems possible.

## Introduction

Monitoring of vessel pressure is of central importance in various clinical fields, such as the evaluation of vital parameters in patients at intensive care units. This is often performed using intraluminal catheters. Thrombosis in conjunction with these catheters is rare (1, 2).

In another clinical field, vessel monitoring is performed by plastic, maxillofacial, or otolaryngological surgeons in conjunction with micro-anastomosis and evaluation of the perfusion of different types of microsurgery flaps. The monitoring is mainly applied on the venous side of the responsible vessels (3).

Conventional free flap monitoring techniques require an external component, whereas an implantable monitor readily indicates changes in free flap perfusion, especially in buried flaps used in head and neck reconstruction. Therefore, different approaches have been described (4).

In fact, two implantable monitoring systems are in clinical use. The first is based on a cough electrode, which circumvents the venous vessel (Cook Swartz doppler probe, Cook Medical, Bloomington, USA). The technological principle of this system is based on a Doppler sensor with an extraluminal placement. It is surgically placed during the suturing of the veins and contains a cable for an external monitoring unit. After a few days, the cable is cut and persists in the local area (5). Different studies have shown that this is a reliable technique for postoperative monitoring (6). Nevertheless, problems were observed related to the removal of the cable. Even dislocated electrodes with failure of blood flow measurement have been described (7).

The second concept is based on an ultrasonic Doppler sensor placed inside a coupler device for end-to-end microsurgical venous anastomosis (Flow coupler, Synovis, Birmingham, USA). The coupler system is intended to detect blood flow and confirm vessel patency intra- and postoperatively at the anastomotic site. The results with this device show a variability in outcome, and these devices are expensive. A comparative study between these two systems observed no significant difference in outcome (8).

**Figure 1:**
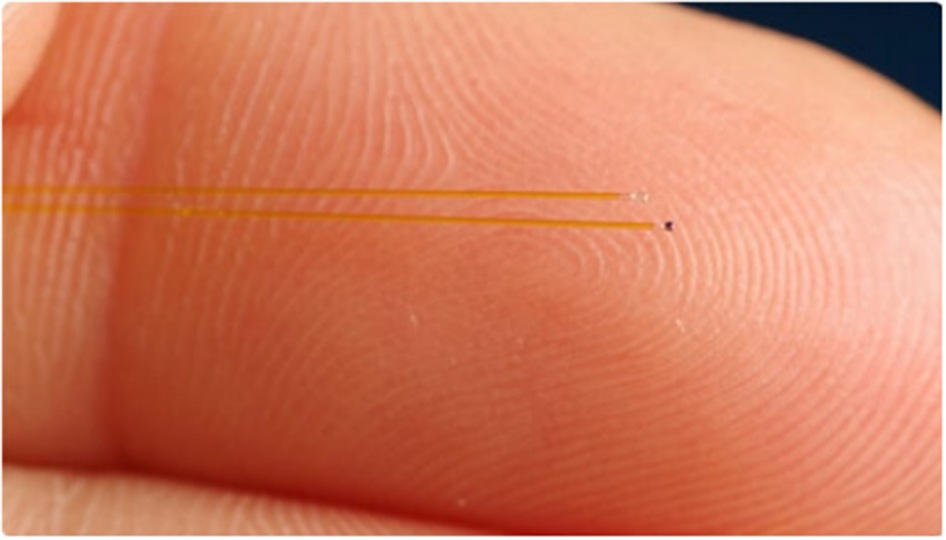
Fiber optic pressure sensor 200 μm (Courtesy of FISO).

Fiber optic sensors are highly accurate and sizeable down to 0.2 mm/ 0.8 Char (FISO, Quebec, Canada). The physical principle of measurements is based on a Fabry-Pérot chamber with a flexible membrane on top and allows measurements at values of ±300 mmHg. The size of the conducting cable is 155 μm, and therefore, it can even be used for conduction out of a very small vessel. The small size and high accuracy of the sensor make it a possible candidate for intraluminal monitoring of vessel pressure. Theoretically, such a system would combine high accuracy related to the intraluminal position and high reliability against dislocations of the sensor itself related to the intraluminal position. Figure 1 is an exemplary picture of a pressure sensor (200 μm).

The aim of the present study was to evaluate, the surgical feasibility of handling, introducing, and using a fiber optic sensor for an intraluminal pressure (ILP) measurement in an animal model of anastomosis.

## Materials and Methods

### Animals

Five female rats and five male Wistar rats (482–616 g) were sedated with a weight-adapted amount of phenobarbital, xylazine, and fentanyl. Then, a surgical approach to the A. carotis communis and V. jugularis was performed on the left side. All experiments were covered by the allowance of the state office (LANUV; file number: 84–02.05.40.16.035).

### Surgical Procedure

According to Simsek et al. (9) the rats were placed in a supine position. The legs were fixed by staples. The head was freely moveable and a surgical access to the left neck was performed. Surgical clips were placed on the A. carotis communis and V. jugularis, and a slot was made in the vessel wall with an 11-blade scalpel (**Figure 4**). The microsensor was introduced (**Figure 5**), and the vessel was closed with Ethilon 10-0 (**Figure 6**). Finally, a drop of fibrin glue was placed on the entrance of the cable. Finally, the clips were removed. The cable was additionally sutured in the surrounding tissue to prevent dislocation. After an hour, we conducted the milking test to evaluate the patency of the vein and arteria distal to the anastomosis (10). Anticoagulant medication was not used.

### Sensor

The ILP was measured using a micro-optical pressure sensor, FOP- MIV (FISO, Quebec, Canada). The tip of the pressure sensor (600 μm) is a hollow glass tube sealed on one end by a thin plastic film diaphragm coated with a reflective surface of evaporated gold. The optical fiber is located in the glass tube, with a short distance (50–100 μm) to the diaphragm tip. The optical fiber is attached to an LED light source and a photodiode sensor. Light from the LED source reaches the sensor tip of the optical fiber, fans out as it exits the fiber, and is reflected by the gold-covered flexible diaphragm. The photodiode senses the reflected light, and small pressure-induced distance displacements of the diaphragm modulate the intensity of the reflected light. The sensor is connected to a module that is linked to a computer. Evolution software was used to record the ILP. The time sensitivity of the sensor was 300 measurements per second.

**Figure 2:**
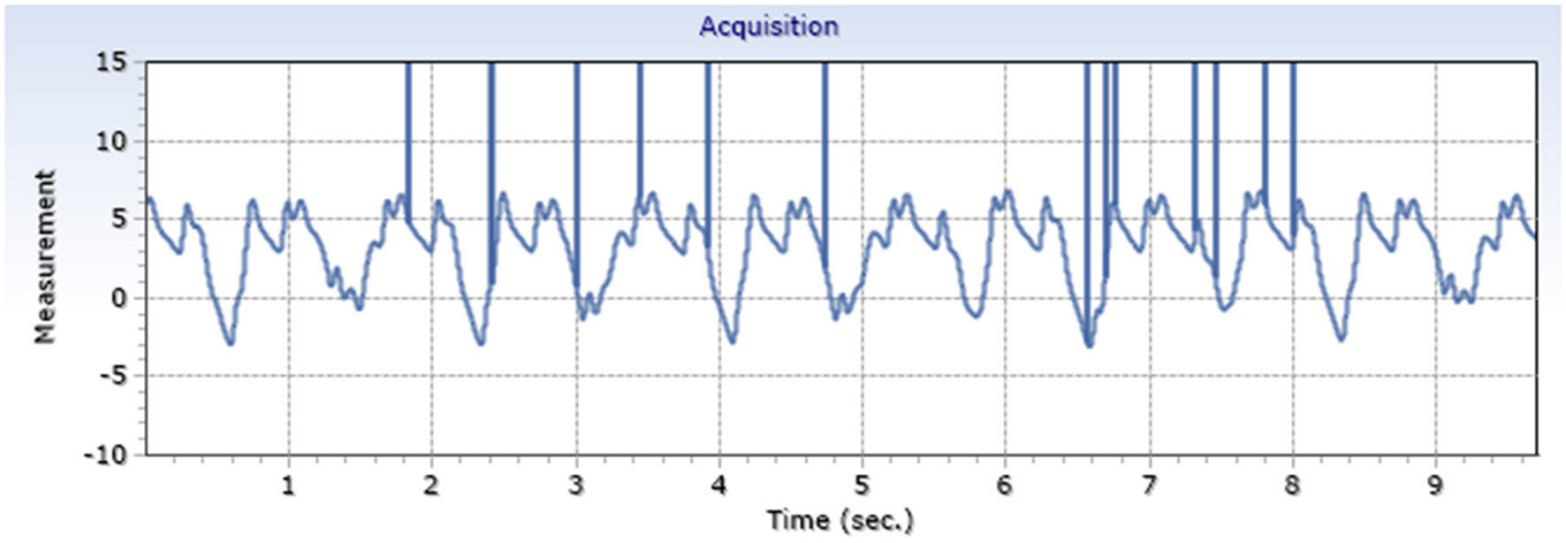
Schematic description of experimental set up. Sensor is introduced in the animal region of interest. Light signal is transferred to the controller and translated into an electrical signal for the laptop.

For a better understanding Figure 2 shows the set up.

## Results

In all cases, introduction of the sensor and closure of the vessel were uneventful and without any leakage around the sensor access. Over the period of measurements, no decrease in the pressure signal was observed indicating a clotting or thrombus around the sensor.

**Figure 3:**
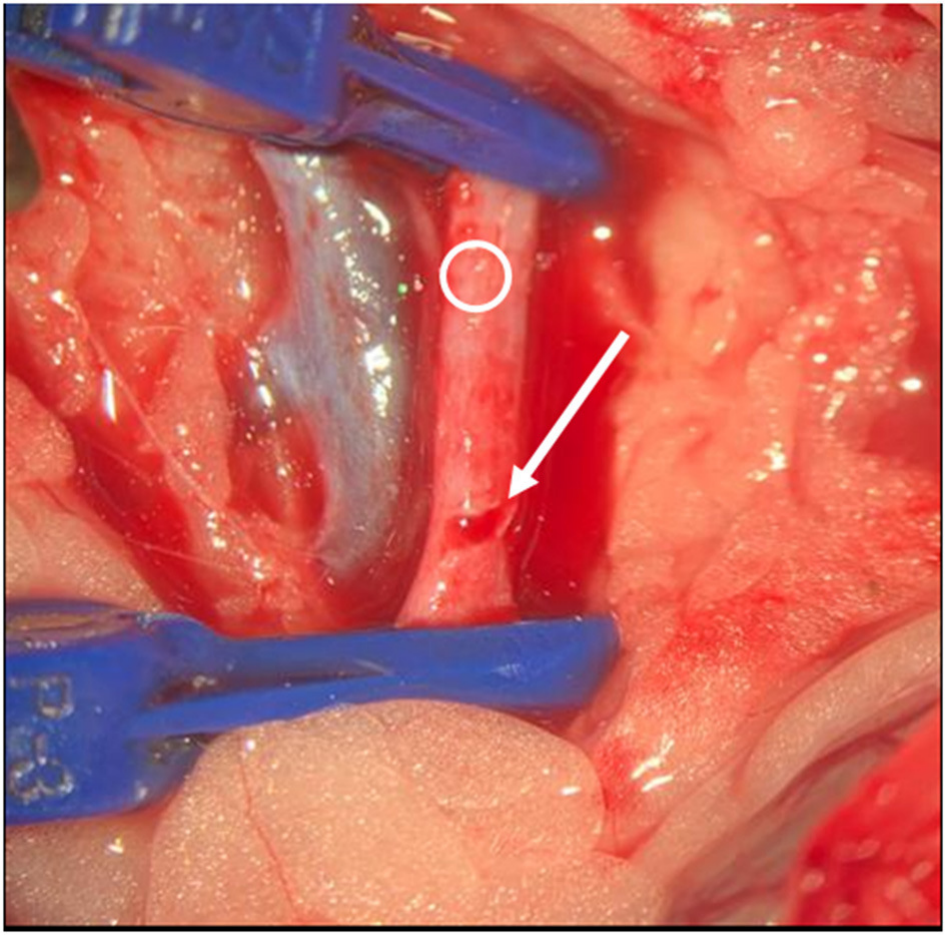
Exemplary arterial pressure signal (A. Carotis) generated by a FOPS x-axis: mmHg, y-axis: time.

**Figure 4:**
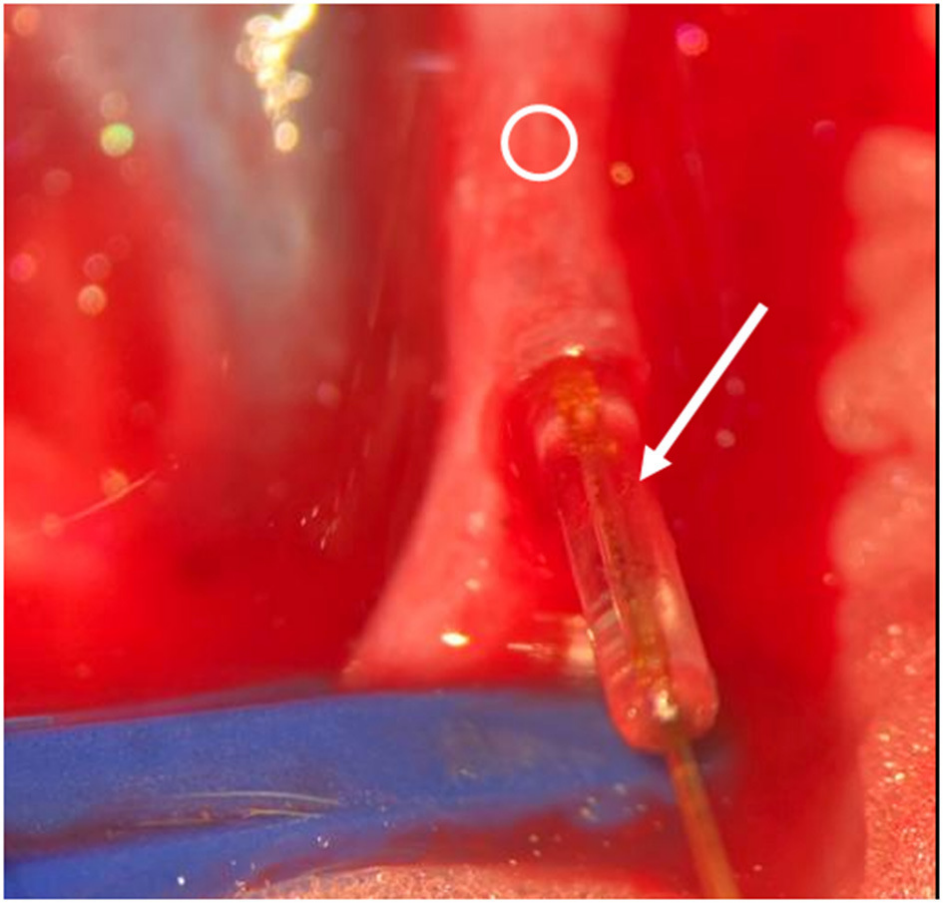
Exemplary venous pressure signal (V. Jugularis) generated by a FOPS x-axis: mmHg, y-axis: time.

**Figure 5:**
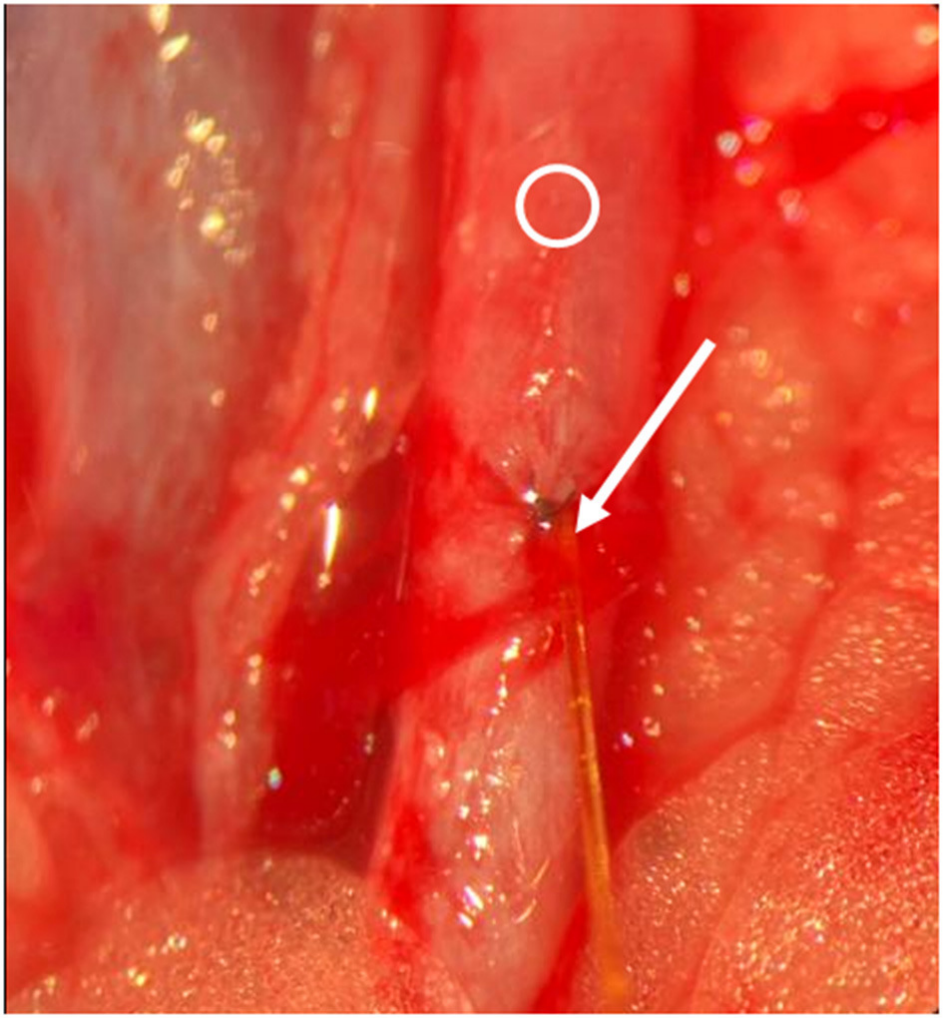
Intraoperative situs. Blue clips are visible, Ring indicates vessel. Arrow indicates slot. Opening of the vessel.

**Figure 6:**
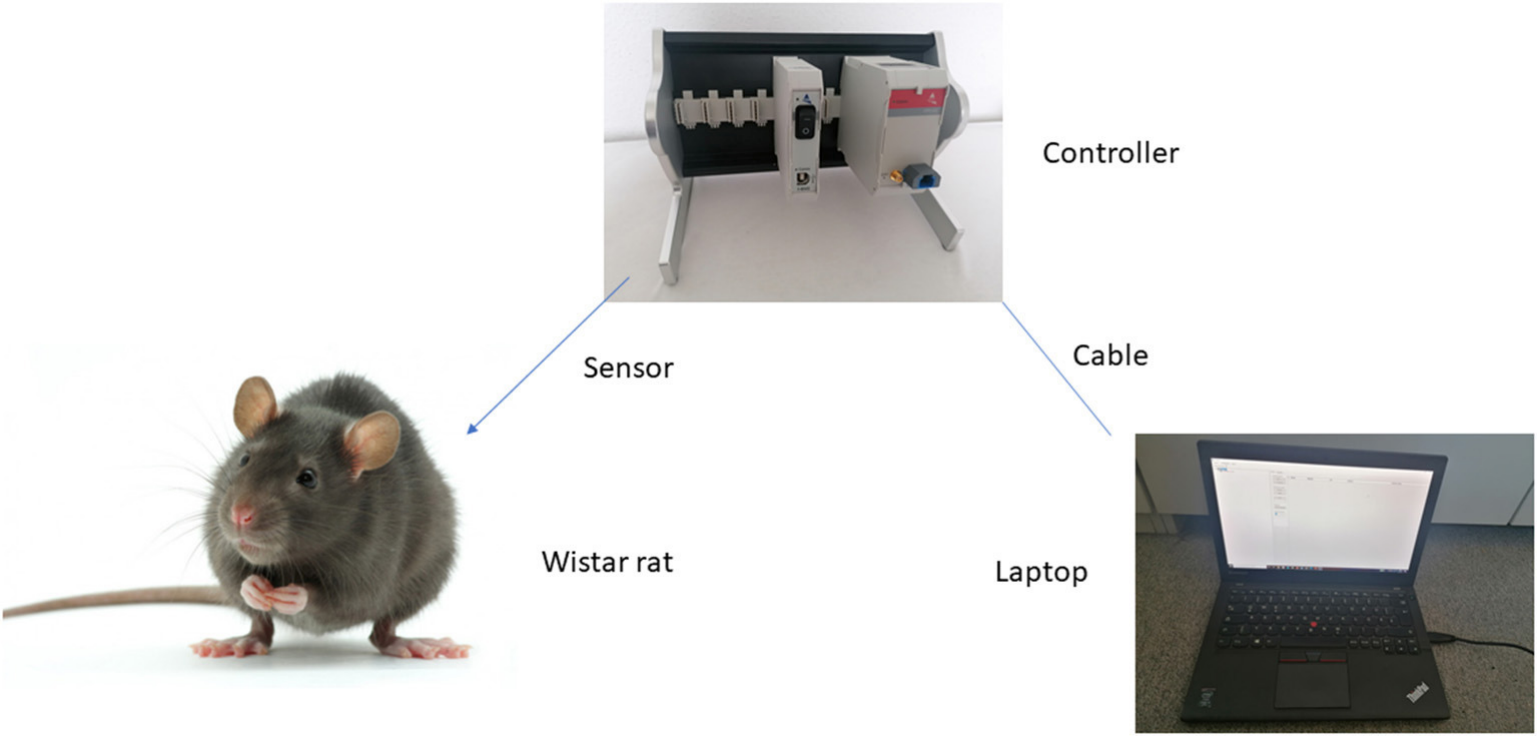
Intraoperative situs. Arrow indicates sensor and cable, Ring indicates vessel. Insertion of the sensor.

**Figure 7:**
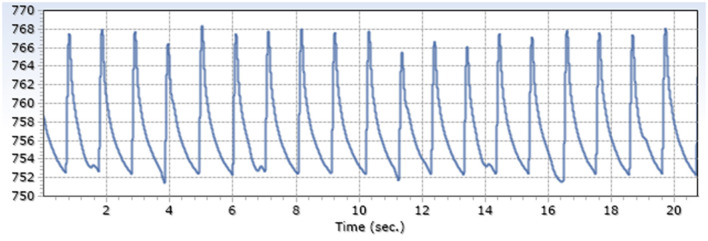
Intraoperative situs. Arrow indicates entrance of the sensor into the vessel, Ring indicates vessel. Sutured vessel with inserted sensor.

Exemplary arterial and venous measurements are shown in Figures 3, 4. Figures 5–7 shows the surgical procedure of introduction of the 600 μm fiber optic pressure sensor (FOPS).

Measured intraluminal mean maximum arterial amplitude was 13.6 mmHg SD 1.9; mean venous amplitude was 5.2 mmHg SD 3.3 (Table 1), with a ratio of 2.6.

**Table 1:** Individual maximum pressure amplitude measured intraluminally by fiber optic pressure sensoring.

	***v1***	***v2***	***v3***	***v4***	***v5***	***V6***	***v7***	***v8***	***v9***	***v10***
**ARTERIAL**
mmHg	15	16	14	15	15	10	12	12	15	12
**VENOUS**
mmHg	2.3	1.8	2	2.1	1.9	7.5	9	8	9	8

*V stands for each animal and the according A. carotis and V. jugularis measurement*.

During our measurement periods (1 h), a stable signal was evaluated, indicating no event of clotting or thrombosis. After an hour, the milking test showed no abnormalities.

## Discussion

Monitoring of vascular anastomosis is a helpful tool to control the perfusion of free flaps. In cases of decreased perfusion, a timely revision can save the flap in some cases. Therefore, monitoring is of high importance. Common monitoring systems, in contrast to visual control of the flap, have the advantage of providing a timely alert before substantial damage to the flap may occur (11).

Current monitoring systems have the disadvantage of a certain degree of false negative or false positive results in terms of alerting the surgeon (12), leading to a search for different systems to improve the monitoring (13, 14). Therefore, systems with a higher accuracy are of high interest. Accuracy is determined by more exact measurement values and a lower degree of sensor dislocation.

FOPSs are a newer form to observe pressure changes based on the principle of a Fabry-Pérot interferometer with a flexible embodiment (15). So far, they have been used in experimental settings to evaluate pressure changes in cardiology (16), neurosurgery (17), and otology (18), as well as for the evaluation of inner ear pressure changes (19).

An application in the field of anastomosis to evaluate microsurgical quality control has not been performed to the best of our knowledge.

Related to its small size and high accuracy, an intraluminal measurement, which eliminates the problem of dislocation of external sensors, seems possible.

Our experiments showed that with microsurgical skills, handling of the fiber optic sensor is easy to perform and was uneventful. Pressure measurements could be performed without any complications.

One limitation of the study was the size of the sensor. The higher dimension of our sensor in contrast to the possible dimensions that could be produced make its use even easier.

Further limitations is that this study is at an very early stage. Further comprehensive animal studies are necessary before a human application can be performed. The important limitations are e.g., the sensors are not approved for human intraoperative use and the fixation of the sensor is an unsolved problem as well as a long term observation is needed.

Although not observed in our measurements, clotting could not be fully excluded. Further observations in a long-term measurement interval would be of high importance.

Surface coating for clotting prevention might solve this theoretical problem (20). Handling of the sensor after the successful control of a persisting anastomosis is a second issue. From our point of view, two options might be chosen: (1). The cable is cut, and the small sensor stays in the vessel; (6) the sensor is pulled out. A 0.2 mm lesion caused by the pullout should be closed immediately as known from the clinical routine with central venous catheters. These options are a matter for further evaluation.

## Conclusion

Fiber optic pressure measurement in microvessels is possible and surgically feasible. An application to monitor the perfusion of free flaps seems possible.

## Data Availability Statement

The original contributions presented in the study are included in the article/supplementary material, further inquiries can be directed to the corresponding author/s.

## Ethics Statement

The animal study was reviewed and approved by LANUV.

## Author Contributions

LW and IT consultant, idea, surgeon, and writing. HS head of Bielefeld ENT and co-writing. OF head of Bielefeld Plastic Surgery and co-writing. All authors contributed to the article and approved the submitted version.

## Conflict of Interest

The authors declare that the research was conducted in the absence of any commercial or financial relationships that could be construed as a potential conflict of interest.
